# Limited Effect of Dopaminergic Medication on Straight Walking and Turning in Early-to-Moderate Parkinson’s Disease during Single and Dual Tasking

**DOI:** 10.3389/fnagi.2016.00004

**Published:** 2016-01-27

**Authors:** Morad Elshehabi, Katrin S. Maier, Sandra E. Hasmann, Susanne Nussbaum, Heinz Herbst, Tanja Heger, Daniela Berg, Markus A. Hobert, Walter Maetzler

**Affiliations:** ^1^Department of Neurodegeneration, Center for Neurology, Hertie Institute for Clinical and Brain Research, University of Tuebingen, Tuebingen, Germany; ^2^German Center for Neurodegenerative Disease (DZNE), Tuebingen, Germany; ^3^Neurozentrum Sophienstrasse, Stuttgart, Germany

**Keywords:** Parkinson’s disease, dual tasking, gait, turning, wearable sensors

## Abstract

**Background:**

In Parkinson’s disease (PD), the effects of dopaminergic medication on straight walking and turning were mainly investigated under single tasking (ST) conditions. However, multitasking situations are considered more daily relevant.

**Methods:**

Thirty-nine early-to-moderate PD patients performed the following standardized ST and dual tasks as fast as possible for 1 min during On- and Off-medication while wearing inertial sensors: straight walking and turning, checking boxes, and subtracting serial 7s. Quantitative gait parameters as well as velocity of the secondary tasks were analyzed.

**Results:**

The following parameters improved significantly in On-medication during ST: gait velocity during straight walking (*p* = 0.03); step duration (*p* = 0.048) and peak velocity (*p* = 0.04) during turning; velocity of checking boxes during ST (*p* = 0.04) and DT (*p* = 0.04). Velocity of checking boxes was the only parameter that also improved during DT.

**Conclusion:**

These results suggest that dopaminergic medication does not relevantly influence straight walking and turning in early-to-moderate PD during DT.

## Introduction

Parkinson’s disease (PD) affects a variety of gait parameters and cognitive functions, such as attention, visuospatial perception, and executive functions (Blin et al., 1991; Dubois and Pillon, 1996; O’Sullivan et al., 1998; Muslimovic et al., 2005). These latter parameters have repeatedly been shown to influence quality of gait (Yogev-Seligmann et al., 2008; Smulders et al., 2013) and may, in particular, influence the quality of turning phases (Stack et al., 2004; McNeely and Earhart, 2011; Song et al., 2012). Due to how neurodegenerative diseases affect daily life activities, turning becomes a frequent and crucial activity that is associated with falls (Bloem et al., 2001; Stack et al., 2004).

Dopaminergic medication has a significant effect on many PD symptoms, with the most relevant improvements found for distal symptoms (Lees et al., 2009). Although not as effective, dopaminergic medication also influences PD-specific straight walking (Blin et al., 1991; Lord et al., 2011; McNeely et al., 2012) and some turning deficits (McNeely and Earhart, 2011). These studies mainly used single tasking (ST) paradigms, which may fall short due to multiple reasons. First, gait is a complex task, involving cognitive elements (Maetzler et al., 2013) Second, dopaminergic medication also influences cognitive functions. Improving some of them yet deteriorating others (Cools et al., 2001, 2003). Finally, ST walking is rarely or never performed during every day situations, since multitasking is performed during virtually all aspects of daily life activities, including walking phases (O’Shea et al., 2002; Hausdorff et al., 2003). Therefore, the influence of dopaminergic medication on straight walking and turning in PD should not only be assessed under ST but also under standardized multitasking situations.

In this study, we focused on the effect of dopaminergic medication on straight walking and turning in ST and dual tasking (DT) conditions.

## Materials and Methods

### Participants

Forty-four PD patients with a Hoehn and Yahr (1967) score between 2 and 3 and a Mini Mental State Examination score >24 (Folstein et al., 1975) were recruited from the ward and the outpatient clinic of the Neurology department, University Hospital Tuebingen. Diagnosis of PD was based on the UK Brain Bank Society criteria (Gibb and Lees, 1988). We included only individuals affected by a mild-to-moderate stage, as these patients have only subtle “subclinical” gait disturbances (Mirelman et al., 2011; Hass et al., 2012) and therefore may be those who benefit most effectively from potential treatment or even preventive options. The response to dopaminergic therapy was evaluated using the motor part of the revised version of the Unified Parkinson’s Disease Rating Scale (UPDRS III) (Goetz et al., 2008) during On- and Off-medication. Off-medication was defined as withdrawal from dopaminergic medication overnight. On-medication condition was defined as 30 min to 2 h after the intake of the participant’s usual dose of dopaminergic medication, considering each study participant’s perception of having a “Good On Phase.” The ethical committee of the medical faculty of the University of Tuebingen approved the study (Nr 715/2011BO2) and all participants provided a written informed consent.

### Tasks

For the assessment of ST straight walking and turning, participants walked as fast as possible up and down a 20-m distance in an at least 2 m wide hallway for 1 min (Mancini, 2011). ST checking boxes and serial subtracting 7s tasks were performed also as fast as possible while standing (crossing 32 empty boxes on a sheet of paper; subtracting a series of ten consecutive steps of 7) (Bock, 2008; Hobert et al., 2011). For the DT assessment, study participants performed the checking boxes and the subtracting serial 7s tasks also for 1 min, respectively, in parallel with the walking task (Brauer and Morris, 2010). For the DT assessments, participants were instructed to perform both tasks as fast as possible.

### Movement Assessment

All participants wore an unobtrusive movement analysis system (Mobility Lab^®^, APDM, OR, USA) (Mancini, 2011) during the tasks. For the analysis presented here, data from the feet and waist sensors were used. For determination of the velocity of the secondary tasks, a stopwatch was used.

### Data Analysis

Based on the previous literature (Plotnik et al., 2007; Lord et al., 2011; Galna et al., 2013), we selected the following six gait parameters to assess the quality of the straight walking phase: gait velocity, step frequency, double limb support time, stride length asymmetry, stride duration variability, and double limb support variability. These parameters represent different domains of walking (pace, variability, and postural control) and have been shown to be relatively independent from each other (Lord et al., 2013). Stride duration variability and double limb support variability were calculated using the SD of the first 30 steps of the straight walk, as previous work (Galna et al., 2013) has shown the reliability of this approach. From the turning phase, we assessed total duration, step duration, number of steps, peak velocity, and the last step duration (Salarian et al., 2009; Hong and Earhart, 2010). Parameters were extracted and organized using Matlab 8.4. On- and Off-medication conditions were compared with paired *t*-test. JMP 11.1.1 statistical software was used. Based on the exploratory character of the study, uncorrected *p* < 0.05 was considered significant.

## Results

From the 44 patients included in this study, 5 showed changes in the UPDRS III scores between 0 and 2 points, which was considered clinically not meaningful (Schrag et al., 2006; Shulman et al., 2010) and led to the exclusion of these patients from further analysis. The remaining 39 PD patients showed an improvement in the score of ≥5 points during On- compared to Off-medication, which was considered clinically significant (Schrag et al., 2006; Shulman et al., 2010). Detailed demographic and clinical data are presented in Table 1.

**Table 1:** **Demographic data of 39 PD patients in On- and Off-medication conditions**.

	Off-medication	On-medication	*p*-Value
Age (years)	65.2 (6.9)		
Gender	M:31; F:8		
MMSE (0–30)		29 (2)	
BDI (0–63)		7 (6)	
UPDRS III (0–132)	30 (9)	21 (7)	<0.0001
Hoehn and Yahr (1–5)	35 patients stage 2		
	4 patients stage 3		

*BDI, Beck Depression Inventory; MMSE, Mini Mental State Examination; UPDRS III, motor part of the Unified Parkinson’s Disease Rating Scale*.

### Effect of Medication on Gait and Turning Parameters during ST and DT

**Figure 1:**
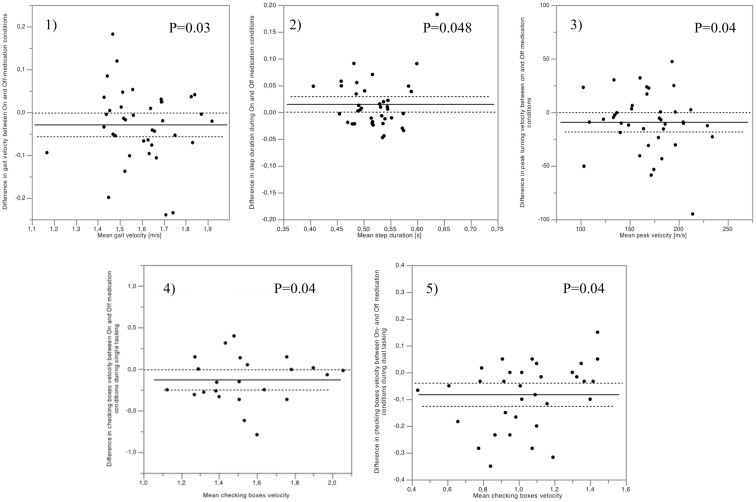
**Significant differences of gait parameters between On- and Off-medication were only observed under single tasking conditions, and affected (1) gait velocity, (2) turning peak velocity, and (3) turning step duration**. Checking boxes velocity was the only parameter significantly affected by medication during single (4) and dual tasking (5). The mean differences for each patient (black dots), as well as mean values (full lines) and SEs (dotted lines) of the cohort are indicated, showing that differences between medication statuses (*y* axes) were not relevantly dependent on the absolute values of the respective parameters (*x* axes).

During ST straight walking, PD patients had significantly higher gait velocity in On- than in Off-medication condition (*p* = 0.03) (Figure 1). Other gait parameters showed no significant difference between the On- and Off-medication conditions. During the ST turning phases, study participants showed significantly lower step duration and higher peak velocity of turning in On- compared to Off-medication condition (*p* = 0.048 and 0.04, respectively) (Figure 1). The rest of the turning parameters showed no significant changes in On- compared to Off-medication conditions.

During both checking boxes and serial subtraction DT settings, no straight walking and turning parameter changed significantly between On- and Off-medication conditions. Details are provided in Table 2.

**Table 2:** **Performances of straight walking, turning, and secondary cognitive tasks in ST and DT conditions**.

Parameter	Off-medication	On-medication	*p*-Value
**Straight walking (ST)**			
Gait velocity (m/s)	1.58 (0.16)	1.61 (0.16)	**0.03**
Step frequency (step/min)	128 (9)	129 (13)	0.25
Double limb support time (%)	16.3 (4.3)	16.2 (4.6)	0.78
Stride length asymmetry (%)	1.60 (0.54)	1.50 (0.53)	0.26
Stride duration variability (SD^a^)	0.03 (0.01)	0.03 (0.01)	0.78
Double limb support variability (SD)	2.2 (0.7)	2.4 (1.0)	0.27
**Straight walking during checking boxes (DT)**			
Gait velocity (m/s)	1.37 (0.17)	1.35 (0.19)	0.18
Step frequency (step/min)	117 (11)	118 (13)	0.49
Double limb support time (%)	19.7 (4.5)	19.4 (4.9)	0.56
Stride length asymmetry (%)	1.87 (0.60)	1.79 (0.66)	0.35
Stride duration variability (SD^a^)	0.04 (0.02)	0.04 (0.02)	0.76
Double limb support variability (SD)	2.8 (1.2)	2.5 (1.1)	0.22
**Straight walking during serial subtraction (DT)**			
Gait velocity (m/s)	1.35 (0.21)	1.37 (0.18)	0.27
Step frequency (step/min)	115 (11)	115 (11)	0.96
Double limb support time (%)	19.1 (4.6)	19.7 (4.2)	0.77
Stride length asymmetry (%)	1.64 (0.59)	1.55 (0.56)	0.34
Stride duration variability (SD^a^)	0.04 (0.03)	0.04 (0.04)	0.90
Double limb support variability (SD)	2.3 (1.6)	2.3 (1.4)	0.69
**Turning (ST)**			
Total duration (s)	2.4 (1.1)	2.2 (0.5)	0.18
Step duration (s)	0.53 (0.05)	0.51 (0.05)	**0.048**
Number of steps (/turn)	5.5 (1.1)	5.3 (1.0)	0.23
Peak velocity (°/s)	164 (34)	173 (37)	**0.04**
Last step duration (s)	0.48 (0.04)	0.48 (0.04)	0.18
**Turning during checking boxes (DT)**			
Total duration (s)	3.3 (1.0)	3.7 (1.5)	0.68
Step duration (s)	0.66 (0.32)	0.63 (0.19)	0.30
Number of steps (/turn)	6.5 (1.6)	6.6 (1.9)	0.90
Peak velocity (°/s)	130 (32)	129 (35)	0.84
Last step duration (s)	0.53 (0.06)	0.53 (0.05)	0.49
**Turning during serial subtraction (DT)**			
Total duration (s)	2.8 (1.1)	2.7 (0.9)	0.41
Step duration (s)	0.60 (0.09)	0.62 (0.20)	0.41
Number of steps (/turn)	5.8 (1.8)	5.4 (1.2)	0.09
Peak velocity (°/s)	147 (42)	154 (40)	0.31
Last step duration (s)	0.53 (0.06)	0.54 (0.08)	0.63
**Secondary tasks**			
Number of checked boxes ST (/min)	89 (16)	98 (16)	**0.04**
Number of checked boxes DT (/min)	60 (16)	65 (14)	**0.04**
Number of serial subtractions ST (/min)	27 (16)	31 (18)	0.51
Number of serial subtractions DT (/min)	21 (11)	22 (11)	0.85

*Values are presented with mean and SD. The paired *t*-test was used for statistical analysis*.

*^a^SD, standard deviation of the first 30 steps from straight walking*. *ST, single tasking, DT, dual tasking*. *The numbers in bold show the significant *p*-values*.

### Effect of Medication on Secondary Cognitive Tasks during ST and DT

For the checking boxes task, the velocity increased significantly during On-medication condition in ST (*p* = 0.04) as well as during DT (*p* = 0.04) compared to Off-medication.

In regard to the serial subtraction test, there was no difference in the velocity of performing the task during ST (*p* = 0.51) or DT (*p* = 0.85) between the On- and Off-medication conditions. For details see Table 2.

## Discussion

The main result of this study with PD patients – including novel aspects such as assessment under challenging conditions, new DT paradigms, inclusion of DT assessment during turns, and analysis of a specific set of quantitative gait parameters – is that dopaminergic medication-induced changes of gait, which are detectable under ST conditions, are not observable under more daily relevant (Silsupadol et al., 2006; Hackney and Earhart, 2010) DT conditions.

In line with previous studies (Baltadjieva et al., 2006; Hong and Earhart, 2010; Lord et al., 2011; Galna et al., 2015), dopaminergic medication improved gait velocity during straight walking under ST conditions. This improvement can certainly have advantages; for example, it makes affected patients able to move faster from one place to another. However, the disadvantage is that other gait parameters obviously do not improve comparably; in fact, none of the other qualitative gait parameters tested in this study was significantly influenced by dopaminergic medication during ST straight walking. This widening gap between faster gait velocity and lack of improvement of variability and postural control associated gait parameters during ST straight walking under dopaminergic On- compared to Off-medication may increase dynamic balance deficits and risk of falling (Boonstra et al., 2008). These findings are in agreement with results described in previous studies. For example, others found that dopaminergic medication does not have a relevant effect on stride time variability (Hausdorff et al., 2003; Lord et al., 2011). Stride time variability has been shown to be associated with attention and cognitive abilities and deteriorates during the course of the disease (Hausdorff et al., 2003; Lord et al., 2011). We also confirmed previous results (Schaafsma et al., 2003; Almeida et al., 2007) where step frequency and double limb support variability were not relevantly influenced by dopaminergic medication during ST straight walking. Therefore, although these latter parameters are obviously affected by PD (Schaafsma et al., 2003; Almeida et al., 2007), the pathological correlate is most probably not of dopaminergic origin. This observation is supported by the results from studies addressing mechanisms of gait impairments in PD patients and how beneficial the medication is to improve them (Blin et al., 1991; Schaafsma et al., 2003; Baker et al., 2008; Rochester et al., 2011, 2012).

Contrary to our results, another study investigating a similar research question (Rochester et al., 2011) detected a significant improvement of gait variability under On-medication. This difference may be best explained by the differences of the designs and protocols used. While the previous study assessed PD patients under convenient speed conditions, we measured under as fast as possible conditions. Based on recent literature, we argue that subtle symptoms, as they are already present in mild-to-moderate PD (Hass et al., 2012), may be detected with higher sensitivity when using challenging conditions (Mirelman et al., 2011; Maetzler et al., 2013; Bergareche et al., 2015).

The influence of dopaminergic medication on ST gait may be higher during turning periods than during straight walking. In agreement with a previous study (McNeely and Earhart, 2011), dopaminergic medication had a significant positive effect on two parameters related to the velocity of turning, i.e., step duration and peak turning velocity. Therefore, comparably to previous studies (Hong and Earhart, 2010; McNeely et al., 2012; Song et al., 2012; Curtze et al., 2015) and also with the results obtained from the ST straight walking assessment, dopaminergic treatment seems to improve the velocity-dependent parameters significantly, but not those parameters that indicate control and timing of turning. Therefore, the main conclusion from the straight walking results may also hold true for ST turning: the widening gap between pace and, e.g., variability measures under dopaminergic On- compared to Off-medication, could be considered negative for the safety of the task. However, studies investigating the effect of medication on turning in PD are scarce and provide conflicting results (Hong and Earhart, 2010; McNeely and Earhart, 2011; McNeely et al., 2012; Curtze et al., 2015). Furthermore, to the best of our knowledge, this study is the first to investigate turning during DT conditions. Eventually, future studies are necessary, considering actually available information and including different turning conditions, to clarify these differences among existing studies conclusively.

As mentioned above, the significant gait differences found during ST were not observed during DT. This finding was not influenced by the nature of the secondary task. As we may perform DT during many of our normal waking state phases, our results suggest a negligible effect of dopaminergic medication on a relevant proportion of walking during everyday situations. If confirmed in future studies, this finding should motivate the development of supportive treatment strategies for PD-associated gait deficits that are independent of dopaminergic medication.

Velocity of checking boxes was the only parameter of this study that significantly improved by dopaminergic medication during both ST and DT. This finding suggests that cognitive tasks with a substantial fine motor aspect, performed with the upper limbs, are stably responsive to dopaminergic medication, regardless whether the task is performed alone or in parallel to other tasks. This finding fits well with the high correlation of the Purdue pegboard test scores with the nigrostriatal dopaminergic deficit as measured with fluorodopa positron emission tomography (Vingerhoets et al., 1997), and with the clinical observation that rigidity and bradykinesia of the upper limbs present with very good responsiveness to dopaminergic treatment.

The study faces some limitations. First, the number of (especially female) participants included in this study is relatively low, and results should therefore be interpreted with caution. However, we did not find relevant differences between male and female performance in a *post hoc* analysis (not shown). Second, our study focused on the assessment of PD patients with relatively early disease stages. Future studies should also include more severely affected patients, to allow generalization of our findings. Finally, due to technical issues, the performance of cognitive tasks was recorded over all gait phases and not separately for straight walking and turning phases. We argue that the results are still ecologically valid, as secondary tasks are routinely performed during walking, e.g., in the home, where straight walking and turning phases regularly alternate.

In conclusion, dopaminergic medication is obviously not relevantly beneficial for mild-to-moderate PD patients during straight walking and turning under DT conditions, which we consider the more daily relevant condition than the usually tested ST condition.

## Author Contributions

ME’s work included the research conceptualization, statistical analysis, and drafting and revising the manuscript. KM contributed by executing the project and by reviewing the manuscript. SH contributed by helping with the project conceptualization, organization, and execution, as well as by reviewing the manuscript. HH contributed to the execution of the project and by reviewing the manuscript. TH and SN contributed to the data acquisition, and by reviewing the manuscript. DB contributed to the research conceptualization and by reviewing the statistical analysis and the manuscript. MH contributed to the project conceptualization, statistical analysis, and manuscript preparation, as well as by reviewing the manuscript. WM work included research design and organization, designing and reviewing the statistical analysis, as well as reviewing the manuscript.

## Conflict of Interest Statement

The authors declare that the research was conducted in the absence of any commercial or financial relationships that could be construed as a potential conflict of interest. The Review Editor Dr. Katarina R. Savic Vujovic declares that, despite being affiliated with the same institution as the Associate Editor Dr. Milica S. Prostran, the review process was handled objectively.
